# Species-dependent susceptibility of enterococci to bovine cathelicidin BMAP-28 and its relationship to membrane fatty acid composition

**DOI:** 10.3389/fmicb.2026.1826834

**Published:** 2026-05-08

**Authors:** Moe Narita, Rintaro Tomioka, Naruhide Miyoshi, Kazumasa Suita, Harutaka Mishima, Emiko Isogai, Jun Xu

**Affiliations:** 1 Department of Animal Microbiology, Graduate School of Agricultural Science, Tohoku University, Sendai, Miyagi, Japan; 2 Department of Bioengineering, School of Engineering, The University of Tokyo, Tokyo, Japan; 3 Department of Bacteriology, Graduate School of Medicine, University of the Ryukyus, Ginowan, Okinawa, Japan

**Keywords:** antimicrobial peptide, cathelicidin, *Enterococcus faecalis*, *Enterococcus faecium*, membrane permeabilization

## Abstract

Antimicrobial peptides (AMPs) are promising agents against multidrug-resistant bacteria. The bovine cathelicidin BMAP-28 is a cationic α-helical AMP with broad-spectrum activity, but its effects on enterococci and determinants of susceptibility remain unclear. Here, we compared the activity of BMAP-28 and four analogs against *Enterococcus faecalis* and *Enterococcus faecium*. *E. fecium* ATCC49533 and *E. faecium* ATCC6059 were used as reference strains. *E. fecium* and *E. faecium* isolates were obtained from the cattle feces. Synthesized BMAP-28 and four analog peptides were used for MIC assay. Killing assays, scanning electron microscopy, LIVE/DEAD staining, fatty acid analysis about membrane composition, and potassium leakage assay were done in this study. *In silico* structural analysis of BMAP-28 was performed for understanding the characteristics of the peptide. *E. faecium* were markedly more susceptible (MIC 520 μg/mL), whereas *E. faecalis* showed high level tolerance (MIC ≥ 80 μg/mL). Killing assays, scanning electron microscopy, and LIVE/DEAD staining consistently showed rapid membrane damage in *E. faecium* but minimal effects in *E. faecalis*. Fatty acid analysis revealed significant differences in membrane composition with *E. faecium* enriched in unsaturated lipids. In contrast, BMAP-28 induced similar potassium leakage from model membranes with different lipid compositions, indicating that lipid differences alone are insufficient to explain the observed species-dependent susceptibility. Together, these results demonstrate pronounced species dependent activity of BMAP against enterococci and suggest that additional cell envelope features beyond lipid composition contribute to the differential response.

## 1 Introduction

Enterococci are Gram-positive commensals of the gastrointestinal tract that have emerged as major opportunistic pathogens in modern healthcare. *Enterococcus* *faecalis* and *Enterococcus faecium* account for most clinical infections, including bacteremia, endocarditis, urinary tract infection, and intra-abdominal infection. Treatment has become increasingly challenging because enterococci readily acquire resistance determinants and display intrinsic tolerance to multiple antibiotic classes (García-Solache and Rice, 2019; Lupia et al., 2022). In particular, multidrug-resistant and vancomycin-resistant *E. faecium* are now prominent causes of hospital-associated infection, highlighting the need for alternative antimicrobial strategies (Arias and Murray, 2012).

Antimicrobial peptides (AMPs) are central components of innate immunity and represent promising leads for next-generation antimicrobials. Many AMPs are cationic and amphipathic, enabling preferential interaction with bacterial envelopes that are rich in anionic surface polymers and phospholipids (Zasloff, 2002). Cathelicidins constitute a widely conserved AMP family in vertebrates and frequently adopt α-helical conformations upon membrane interaction (Zanetti, 2004; Porcelli et al., 2008). Their antibacterial activity often involves rapid disruption of cytoplasmic membrane integrity, leading to depolarization, permeabilization, and cell death. However, AMP efficacy can vary substantially between bacterial species and even between closely related taxa, reflecting differences in envelope architecture and lipid composition (Yeaman and Yount, 2003).

BMAP-28 is a bovine cathelicidin-derived peptide reported to display broad antibacterial activity, including against Gram-positive pathogens (Brogden et al., 2007). Despite this, comparatively little is known about its activity against enterococci and, importantly, the bacterial determinants that govern susceptibility. Enterococci possess multiple mechanisms that can reduce AMP activity, including cell-envelope remodeling that alters surface charge and membrane biophysical properties (Fabretti et al., 2006). Beyond electrostatic effects, membrane lipid composition, particularly fatty-acid saturation and chain composition can influence membrane order, fluidity, and the energetic cost of peptide insertion, and has been linked to AMP resistance or tolerance in several Gram-positive bacteria (Kumariya et al., 2015). These considerations suggest that differential membrane properties between *E. faecalis* and *E. faecium* could contribute to species-specific responses to BMAP-28.

In this study, we quantified the antimicrobial activity of BMAP-28 and four designed analogs against *E. faecalis* and *E. faecium* using broth microdilution and short-time killing assays and examined membrane damage by scanning electron microscopy and LIVE/DEAD fluorescence staining. To explore whether membrane composition is associated with susceptibility, we compared cellular fatty-acid profiles by gas chromatography of fatty acid methyl esters. We further evaluated membrane permeabilization in a simplified model system by measuring potassium leakage from phosphatidylcholine/phosphatidylglycerol large unilamellar vesicles formulated to mimic enterococcal lipid features (Silberstein et al., 1999). Finally, we incorporated in silico structural analysis to provide a conformational framework for BMAP-28 membrane activity. Together, these experiments address whether BMAP-28 exhibits species-dependent activity against enterococci and identify membrane-level features that may contribute to the differential response of *E. faecalis* and *E. faecium* to this cathelicidin.

## 2 Materials and methods

### 2.1 Bacterial strains and growth conditions

*E. faecalis* ATCC49533 and *E. faecium* ATCC6059 were used as reference strains (Nasr Azadani et al., 2020). Additional *E. faecalis* and *E. faecium* isolates were included where indicated for minimal inhibitory concentration (MIC) range determination. Additional isolates (*n* = 6 per species) were obtained from the cattle feces (Charles Mubita, 2008). Species identification was performed using matrix-assisted laser desorption/ionization time-of-flight mass spectrometry (MALDI-TOF MS; Bruker Daltonics, Bremen, Germany). Antimicrobial susceptibility profiles were determined by broth microdilution according to Clinical and Laboratory Standards Institute (CLSI) guidelines. Unless otherwise stated, strains were cultured in Brain Heart Infusion (BHI) broth at 37 °C for 18-19 h. For assays requiring mid-exponential phase cells, overnight cultures were diluted into fresh BHI and grown to OD_660_ = 0.5 at 37 °C.

### 2.2 Peptides

**Figure 1:**
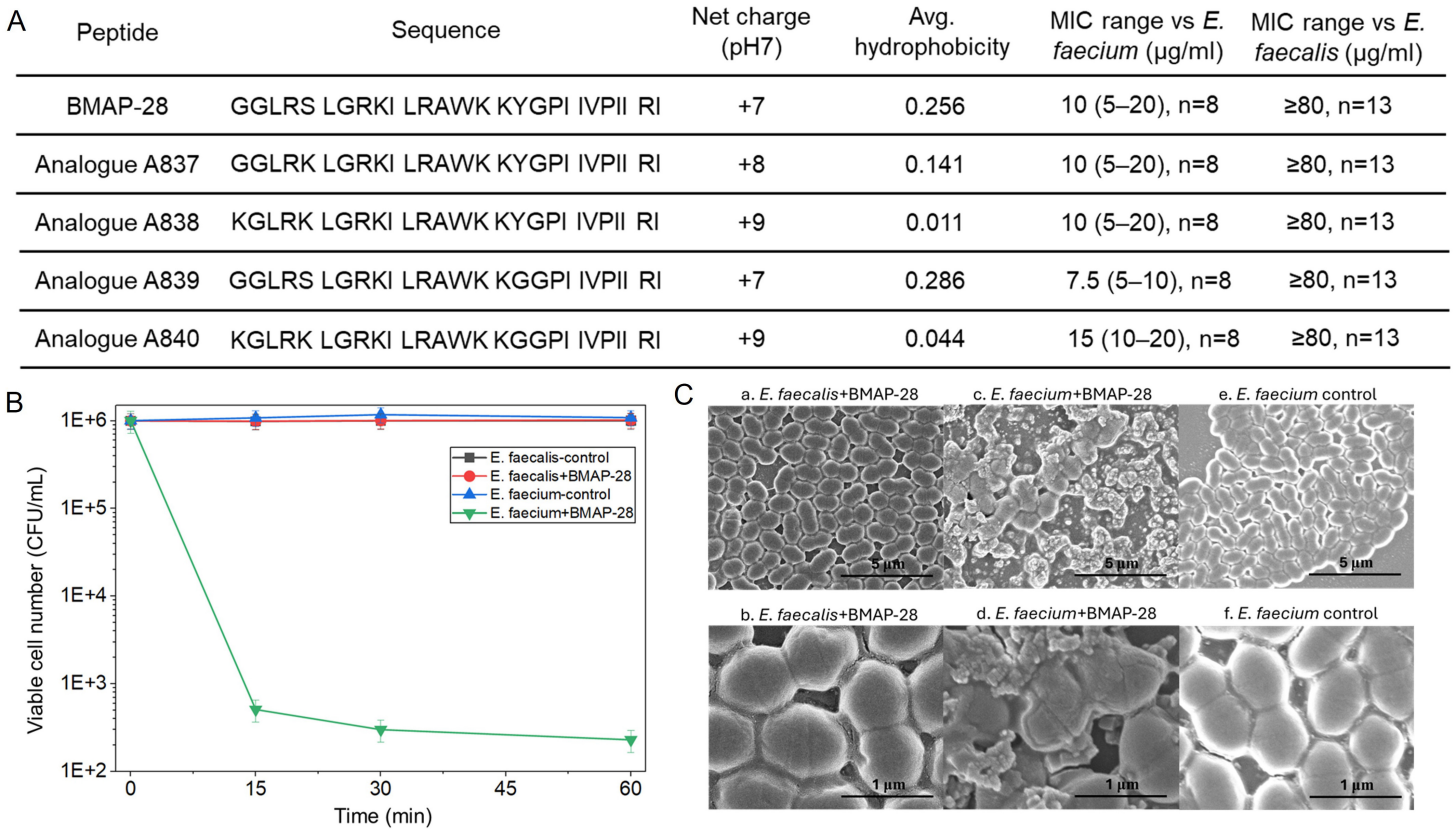
Species-dependent antimicrobial activity of BMAP-28 against enterococci and lack of susceptibility shift by analog design. **(A)** Median MIC values (range) of BMAP-28 and four analogs against *E. faecium* and *E. faecalis*, determined by broth microdilution. **(B)** Short-time killing assay showing viable cell numbers (CFU/mL; logarithmic scale) of *E. faecium* and *E. faecalis* under control conditions or after treatment with BMAP-28 for the indicated times. Data are presented as mean ± SD. **(C)** Scanning electron microscopy (SEM) images of enterococci after exposure to BMAP-28 or control conditions. *E. faecalis* treated with BMAP-28 (a, b), *E. faecium* treated with BMAP-28 (c, d), and untreated *E. faecium* control (e, f). Top row shows lower magnification (scale bar, 5 μm) and bottom row shows higher magnification (scale bar, 1 μm).

BMAP-28 and four analog peptides (A837, A838, A839, and A840) were synthesized, purified, and characterized by KNC Laboratories Co., Ltd (Kobe, Japan) and Peptide Institute Inc. (Osaka, Japan) as previously described (Isogai et al., 2009; Takagi et al., 2014). The peptides were purified by reverse-phase high-performance liquid chromatography (HPLC, Model LC-8A, Shimazu Co., Kyoto, Japan) on YMC-A 302 column (YMC Co., Ltd, Kyoto, Japan) and verified by electrospray ionization mass spectrometry (ESI-MS) using an LCMS-2020 system (Shimadzu, Kyoto, Japan), with all peptides exhibiting the expected molecular masses and a purity exceeding 96.5%. Peptides were supplied as trifluoroacetate salts, dissolved in Hanks' balanced salt solution (HBSS) (pH 7.4) or phosphate buffered saline (PBS) to prepare stock solutions, aliquoted, and stored at −20 °C. Working solutions were prepared by dilution in the appropriate assay buffer immediately prior to use. Peptide sequences and calculated physicochemical properties are summarized in Figure 1C.

### 2.3 MIC determination and short-time killing assay

MICs were determined by broth microdilution in 96-well plates. Mid-exponential phase cultures were diluted in BHI to a final concentration of 1-5 × 103 CFU/mL. Peptides were prepared as two-fold serial dilutions in Brain heart infusion (BHI) to final concentrations of 1.25–80 μg/mL. Bacterial suspension (100 μL) was mixed with peptide solution (100 μL) in each well and incubated overnight at 37° C. Optical density was measured at 660 nm. MIC was defined as the lowest peptide concentration yielding OD_660_ < 0.2, with growth controls typically exceeding OD_660_ > 1.2. Short-time killing assays were performed using mid-exponential phase cells. Bacterial suspensions were adjusted to 1-5 × 108 CFU/mL and distributed into 96-well plates (100 μL per well). BMAP-28 was added (100 μL per well) to a final concentration of 100 μg/mL (corresponding to 5 × MIC under these conditions). Plates were incubated at 37 °C. At 0, 15, 30, and 60 min, aliquots (20 μL) were removed, serially diluted, and plated on BHI agar. Plates were incubated overnight at 37 °C and viable counts were expressed as CFU/mL.

### 2.4 Scanning electron microscopy (SEM)

Mid-exponential phase cultures were adjusted to 1–5 × 108 CFU/mL in Dulbecco's phosphate-buffered saline (DPBS) and treated with BMAP-28 at 5 × MIC for 1 h at 37° C. Cells were then fixed with 2.5% (v/v) glutaraldehyde in 0.1 M phosphate buffer (pH 7.4) for 2 h at room temperature, followed by washing three times with the same buffer. Samples were post-fixed with 1% (w/v) osmium tetroxide for 1 h, washed, and dehydrated through a graded ethanol series (30%, 50%, 70%, 80%, 90%, 95%, and 100%). Dehydrated samples were dried using a critical point dryer, mounted on aluminum stubs, and sputter-coated with a palladium alloy. Imaging was performed using a field-emission scanning electron microscope (SU5000, Hitachi High-Technologies Corporation, Tokyo, Japan) at an accelerating voltage of 5 kV.

### 2.5 LIVE/DEAD membrane permeabilization assay

Membrane integrity of *E. faecium* was assessed using the LIVE/DEAD BacLight bacterial viability assay (Thermo Fisher Scientific) according to the manufacturer's instructions. Cells were incubated in PBS (negative control), with BMAP-28 (20 μg/mL), or with 70% (v/v) isopropanol (positive control) for 1 h at 37 °C, followed by staining with SYTO 9 and propidium iodide (PI) for 15 min in the dark at room temperature. Stained cells were imaged using a fluorescence microscope (Eclipse Ci-L, Nikon, Tokyo, Japan) equipped with appropriate filter sets for SYTO 9 (excitation/emission: 480/500 nm) and PI (535/617 nm). Images were acquired with a CMOS camera (DFK-33UX273, The Imaging Source, Germany) under identical exposure settings for all conditions. For quantitative analysis, at least five randomly selected fields per sample were analyzed using ImageJ (NIH). Cells were classified as PI-positive when the red fluorescence intensity exceeded a threshold defined from PBS-treated control samples (mean + 2 standard deviations of background signal). The percentage of membrane-compromised cells was calculated as the number of PI-positive cells divided by the total number of cells.

### 2.6 Extraction and GC analysis of cellular fatty acids (FAMEs)

Total cellular lipids were extracted using a chloroform–methanol method and polar lipids were enriched using an aminopropyl solid-phase extraction cartridge (Kim and Salem, 1990). Fatty acids were converted to fatty acid methyl esters (FAMEs) and analyzed by gas chromatography with flame ionization detection. Peaks were identified by comparison to a commercial FAME standard mixture. Relative abundance of major fatty acids was calculated from integrated peak areas and expressed as a percentage of the total area of the six major peaks (C12:0, C16:0, C16:1, C18:0, C18:1, and C20:4), as shown in Figure 3C.

### 2.7 Preparation of potassium-loaded large unilamellar vesicles and potassium leakage assay

Large unilamellar vesicles (LUVs) were prepared from phosphatidylcholine (PC) and phosphatidylglycerol (PG) at a molar ratio of 7:3. The PC component was 1-palmitoyl-2-oleoyl-sn-glycero-3-phosphocholine (POPC), which was kept identical across all vesicle formulations. Two PG species were used to model enterococcal lipid differences: 1,2-distearoyl-sn-glycero-3-phosphoglycerol (DSPG) and 1-stearoyl-2-oleoyl-sn-glycero-3-phosphoglycerol (SOPG). DSPG was used as a representative fully saturated PG species to provide a clear contrast with the partially unsaturated SOPG, although it does not exactly reflect the dominant C16:0 fatty acid species observed in enterococcal membranes.

Lipids dissolved in chloroform were dried to form thin films under a stream of nitrogen and further desiccated under vacuum for >1 h. The lipid films were hydrated with 150 mM KCl and sonicated for 6 min at 70° C to obtain vesicles. Vesicles were cooled to room temperature and stored at 4° C overnight prior to use.

K^+^-loaded LUVs were washed three times with PBS to remove external potassium and resuspended in 1 mL PBS. LUVs were incubated with BMAP-28 at final peptide concentrations of 20, 40, or 80 μg/mL. Maximum leakage was defined by addition of Triton X-100 to a final concentration of 2.0% (v/v). Potassium concentrations were measured using an ion-selective electrode (Silberstein et al., 1999). Percent leakage was calculated as:


$$\begin{array}{l} \text{Leakage }\left(\%\right)=100\times \frac{K-K_{0}}{K_{t}-K_{0}} \end{array}$$

Where, *K* is potassium measured after peptide treatment, *K*_0_ is the potassium level in the absence of peptide, and *K*_*t*_ is potassium after Triton X-100 treatment.

### 2.8 *In silico* structural analysis

Homology models of BMAP-28 were generated using cathelicidin templates and subjected to molecular dynamics simulations at 300 K for 10 ns with sampling every 1 ps. Trajectories were clustered by RMSD to identify representative conformational motifs (extended helix, folded into two parts, and compact folded helix) (Bussi et al., 2007). Secondary structure was analyzed across the sequence to compute per-residue helix occupancy, and regions with consistently high helicity and hinge-like flexibility were identified (Figure 5).

### 2.9 Statistical analysis

Unless otherwise stated, data are presented as mean ± SD. Statistical significance was assessed using two-sided tests as appropriate for each dataset. A *p* value < 0.05 was considered statistically significant.

## 3 Results

### 3.1 BMAP-28 shows strong species-dependent antimicrobial activity against enterococci, and analog modifications do not overcome *E. faecalis* tolerance

We first compared the activity of BMAP-28 against *E. faecalis* and *E. faecium* and evaluated four designed analogs with altered net charge and/or hydrophobicity (Figure 1A). BMAP-28 inhibited *E. faecium* with MICs in the range of 5-20 μg/mL, whereas *E. faecalis* exhibited high-level tolerance with MICs of ≥80 μg/mL (Figure 1A). The analogs retained activity against *E. faecium* (MICs typically 5-20 μg/mL) but did not reduce the MIC for *E. faecalis* (all ≥80 μg/mL; Figure 1A). Antimicrobial susceptibility testing of the isolates did not reveal any clear relationship between vancomycin susceptibility profiles and BMAP-28 activity under the conditions tested (Data not shown). Thus, within the physicochemical space explored here (net charge +7 to +9; average hydrophobicity 0.011-0.286), the analog designs did not overcome the pronounced species difference in susceptibility.

### 3.2 BMAP-28 rapidly kills *E. faecium* but not *E. faecalis* in short-time killing assays

To determine whether the MIC differences reflected differences in bactericidal activity, we performed short-time killing assays (Figure 1B). Under control conditions, viable counts of both species remained near 106 CFU/mL over 60 min. Upon BMAP-28 exposure, *E. faecium* showed rapid killing with a drop from ~106 CFU/mL at time 0 to approximately 10^2^-103 CFU/mL by 15 min, remaining near this level through 60 min (Figure 1B). In contrast, *E. faecalis* showed minimal reduction in CFU/mL over the same time course and remained close to the condition of control (Figure 1B). These data demonstrate a strong bactericidal effect of BMAP-28 against *E. faecium*, whereas *E. faecalis* is largely refractory under the conditions tested.

### 3.3 SEM reveals severe envelope disruption in BMAP-28–treated *E. faecium*

To test whether rapid killing correlated with visible cell envelope damage, we examined peptide-treated cells by SEM (Figure 1C). *E. faecium* exposed to BMAP-28 displayed extensive morphological disruption at both low and high magnification, including pronounced surface deformation consistent with severe envelope damage (Figure 1C). In contrast, untreated *E. faecium* maintained smooth, intact morphology (Figure 1C). Under the same treatment, *E. faecalis* showed substantially less overt disruption relative to the changes observed for *E. faecium* (Figure 1C). Together, these observations support that susceptibility of *E. faecium* to BMAP-28 is associated with marked envelope damage.

### 3.4 BMAP-28 causes extensive membrane permeabilization of *E. faecium* as measured by LIVE/DEAD staining

**Figure 2:**
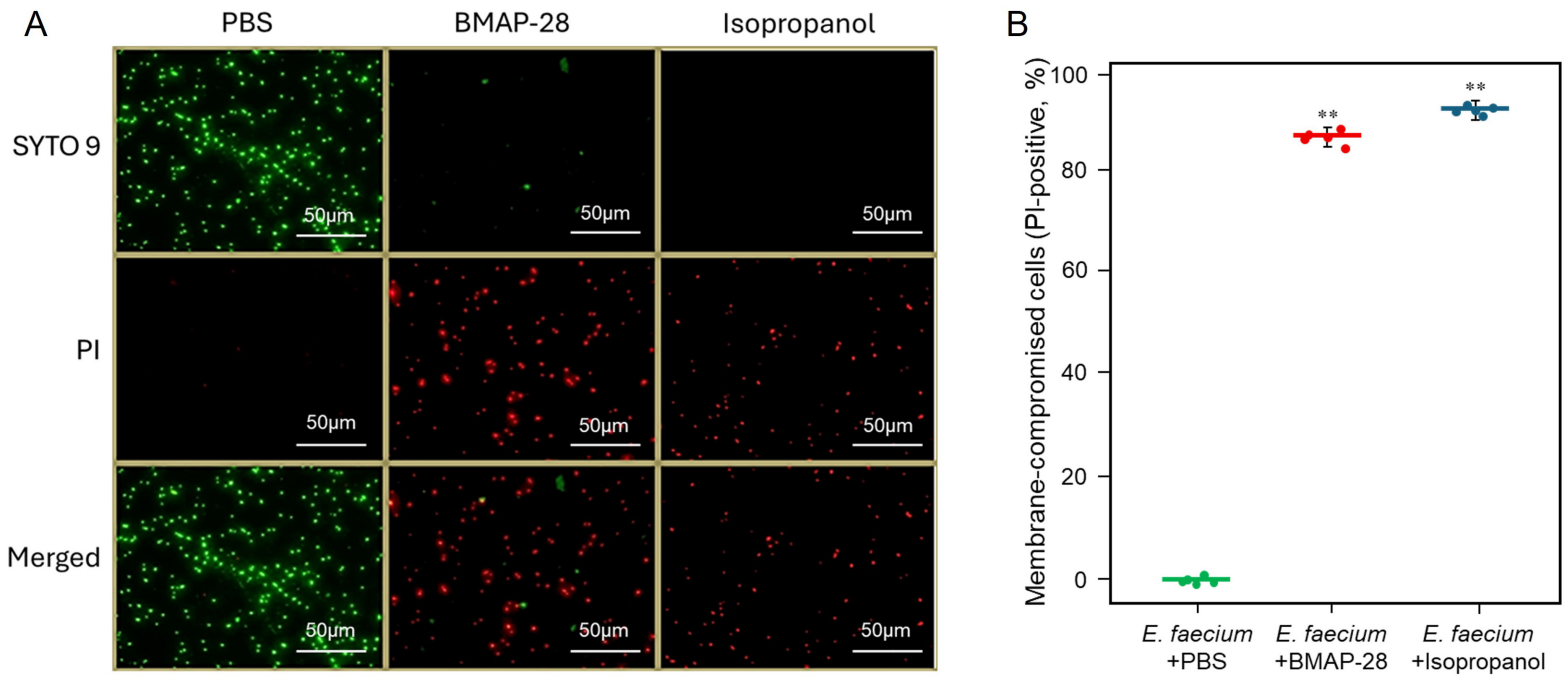
BMAP-28 causes rapid membrane permeabilization of *E. faecium* as assessed by LIVE/DEAD staining. **(A)** Representative fluorescence micrographs of *E. faecium* stained with SYTO 9 (green; membrane-intact cells) and propidium iodide (PI; red; membrane-compromised cells) after incubation in PBS (negative control), with BMAP-28, or with isopropanol (positive control). Images show the SYTO 9 channel (top), PI channel (middle), and merged images (bottom). Scale bars, 50 μm. **(B)** Quantification of membrane-compromised cells expressed as the percentage of PI-positive cells. Cells were classified as PI-positive based on a fluorescence intensity threshold determined from PBS-treated controls (mean + 2 SD). Points represent individual fields of view, and horizontal bars indicate mean ± SD (n = 5). ^**^ indicates *p* < 0.01 compared with PBS control.

We next quantified membrane permeabilization using SYTO 9/propidium iodide (PI) LIVE/DEAD staining in *E. faecium* (Figure 2). PBS-treated cells were predominantly SYTO 9–positive with little PI signal, indicating intact membranes (Figure 2A). In contrast, BMAP-28 treatment markedly increased PI staining, consistent with widespread membrane compromise (Figure 2A). Isopropanol served as positive control and similarly produced strong PI staining (Figure 2A). Quantification of PI-positive cells confirmed that membrane-compromised cells increased from near-baseline levels in PBS to ~85-90% following BMAP-28 treatment and to ~90-95% following isopropanol treatment (Figure 2B). Both BMAP-28 and isopropanol conditions were significantly increased relative to PBS (*p* < 0.01, as indicated in Figure 2B). These data indicate that BMAP-28 rapidly permeabilizes *E. faecium* membranes, consistent with a membrane-disruptive mode of action.

### 3.5 *E. faecalis* and *E. faecium* display distinct membrane fatty acid compositions

**Figure 3:**
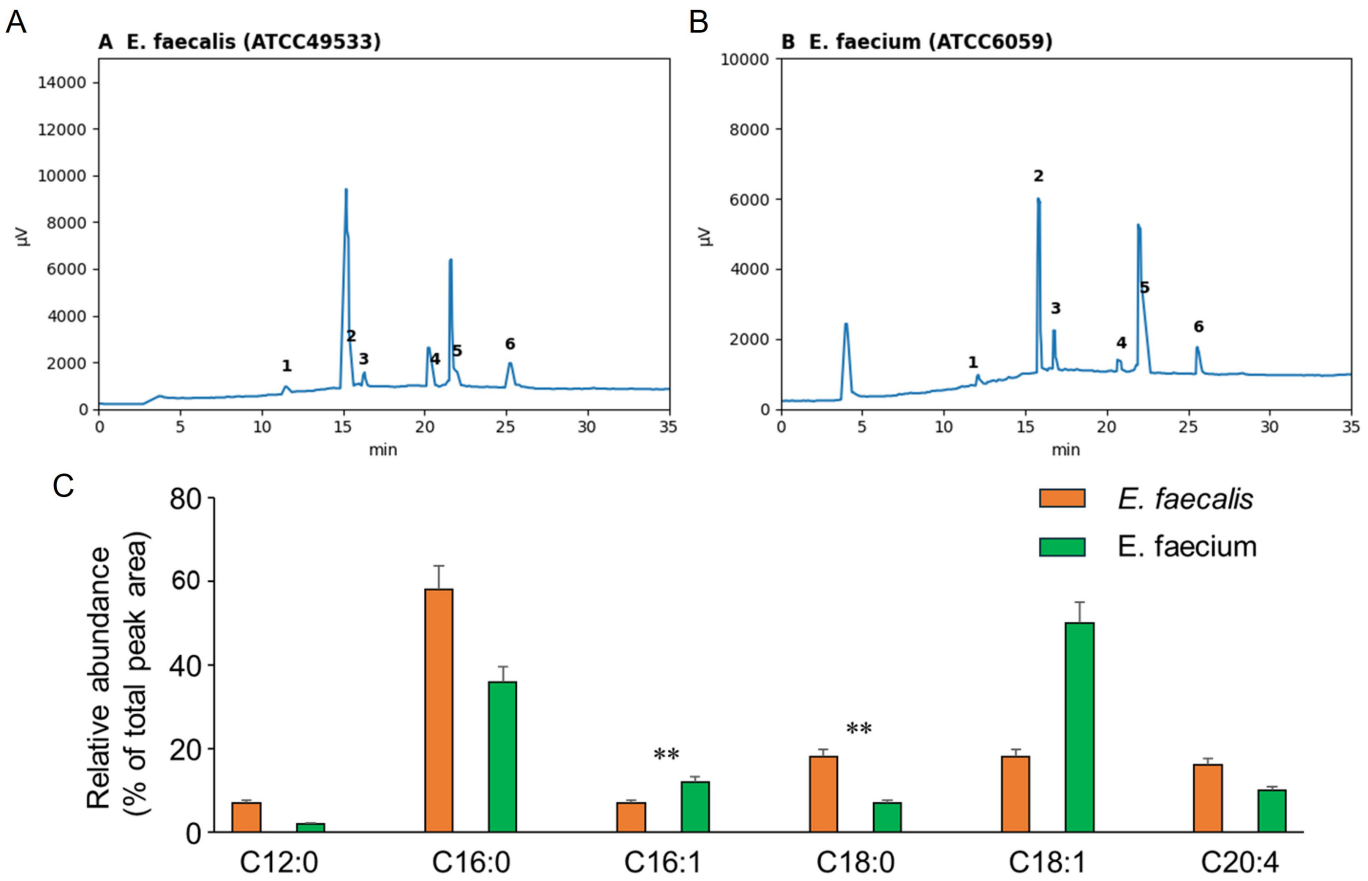
Enterococcal species display distinct membrane fatty acid profiles. **(A, B)** Representative gas chromatograms of fatty acid methyl esters (FAMEs) prepared from membrane lipids of **(A)** *E. faecalis* ATCC49533 and **(B)** *E. faecium* ATCC6059. Numbered peaks correspond to the following fatty acids: (1) C12:0, (2) C16:0, (3) C16:1, (4) C18:0, (5) C18:1, and (6) C20:4. **(C)** Relative abundance of major fatty acid species calculated from integrated peak areas and expressed as percentage of the total area of the six indicated peaks. Bars represent mean ± SD from n = 3 independent cultures per species.

Given the strong species difference in membrane disruption, we next compared cellular fatty acid profiles of *E. faecalis* and *E. faecium* by GC analysis of fatty acid methyl esters (FAMEs) (Figure 3). Representative chromatograms identified six major fatty acids under our conditions-C12:0, C16:0, C16:1, C18:0, C18:1, and C20:4-present in both species (Figures 3A, B). However, their relative abundances differed substantially (Figure 3C).

Quantification showed that *E. faecalis* had a higher proportion of saturated fatty acids (notably C16:0 and C18:0), whereas *E. faecium* was enriched in unsaturated fatty acids (particularly C18:1) and showed a higher proportion of C16:1 (Figure 3C). Differences were statistically significant for C16:1 and C18:0 (Figure 3C; *p* < 0.01). These results demonstrate species-specific fatty acid compositions in enterococci that may influence membrane physicochemical properties relevant to BMAP-28 susceptibility.

### 3.6 BMAP-28 induces potassium leakage from PG-containing model membranes with minimal dependence on lipid composition

**Figure 4:**
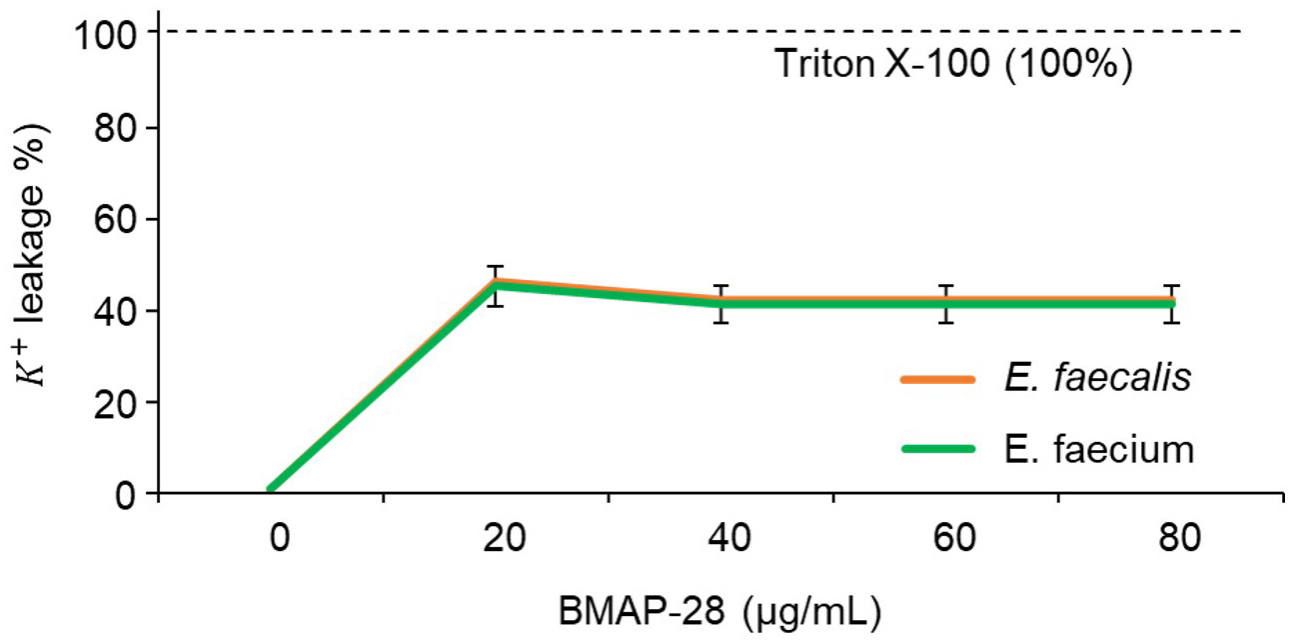
BMAP-28 induces potassium leakage from phosphatidylglycerol-containing model membranes regardless of enterococcal lipid-type mimic. Potassium (K^+^) leakage from K^+^-loaded large unilamellar vesicles (LUVs) after incubation with increasing concentrations of BMAP-28 (0–80 μg/mL). LUVs were prepared using phosphatidylcholine (PC) and phosphatidylglycerol (PG) lipid compositions selected to mimic the major membrane fatty-acid characteristics of *E. faecalis* (orange) or *E. faecium* (green). K^+^ release is expressed as percentage of the maximal leakage induced by Triton X-100 (dashed line; 100%). Data are shown as mean ± SD.

To examine whether membrane lipid composition alone can account for the observed species-dependent susceptibility, we assessed peptide-induced permeabilization using potassium-loaded large unilamellar vesicles (LUVs) composed of phosphatidylcholine and phosphatidylglycerol (PC/PG). Vesicles were formulated to approximate key fatty acid characteristics of *E. faecalis* and *E. faecium* membranes (Figure 4). BMAP-28 induced substantial K? leakage at 20 μg/mL, reaching approximately 45-50% of the Triton X-100 maximum, and leakage plateaued at higher concentrations (40-80 μg/mL) (Figure 4). Notably, leakage profiles were highly similar between the two LUV compositions across all tested concentrations (Figure 4).

These results indicate that, within the simplified PC/PG model system used here, the membrane-permeabilizing activity of BMAP-28 is robust and not strongly dependent on the differences in lipid composition modeled in this study. Thus, lipid composition alone, as represented in this system, is insufficient to explain the pronounced species-dependent susceptibility observed in intact cells. Instead, these findings suggest that additional features of the native enterococcal envelope, including more complex lipid organization and cell envelope components, likely contribute to the differential response to BMAP-28.

### 3.7 *In silico* analysis indicates BMAP-28 forms a predominantly α-helical peptide with hinge regions enabling multiple conformations

**Figure 5:**
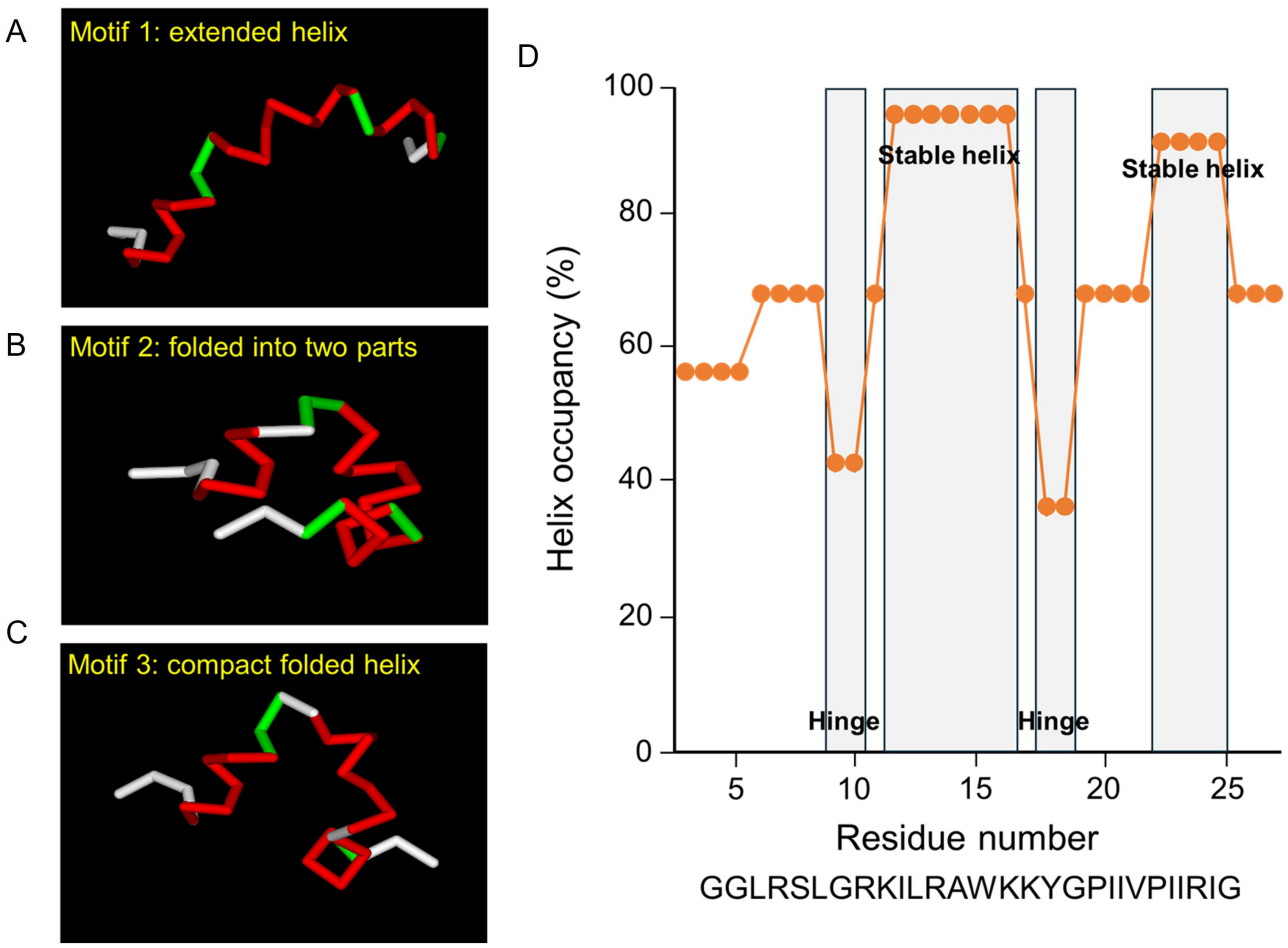
In silico structural analysis reveals that BMAP-28 forms a predominantly α-helical peptide with hinge regions that enable multiple conformations. **(A–C)** Representative conformers of BMAP-28 obtained from homology modeling followed by molecular dynamics simulations, illustrating three major structural motifs observed across simulations: **(A)** an extended α-helical conformation (Motif 1), **(B)** a conformation folded into two parts (Motif 2), and **(C)** a compact folded helical conformation (Motif 3). **(D)** Secondary-structure summary along the BMAP-28 sequence showing helix occupancy (percentage of simulation frames assigned as α-helix for each residue) plotted against residue number. Regions consistently adopting α-helical structure (“stable helix”) correspond to Ala12-Lys16 and Pro23-Ile25, whereas reduced helicity at Lys9-Ile10 and Gly18-Pro19 indicates hinge-like segments that contribute to peptide bending/folding. The BMAP-28 sequence is shown below the plot for reference.

To provide a structural framework for membrane disruption by BMAP-28, we incorporated in silico structural analysis based on homology modeling and molecular dynamics simulations (Figure 5). Three major conformer motifs were observed: an extended helix (Motif 1), a conformation folded into two parts (Motif 2), and a compact folded helix (Motif 3) (Figures 5A–C). Secondary-structure analysis along the sequence showed regions with consistently high helix occupancy (“stable helix”) corresponding to Ala12-Lys16 and Pro23-Ile25, whereas reduced helicity at Lys9-Ile10 and Gly18-Pro19 indicated hinge-like segments that contribute to bending/folding (Figure 5D). These features support a model in which BMAP-28 behaves as an amphipathic α-helical peptide with intrinsic flexibility, consistent with its strong membrane permeabilization activity observed in both cells (Figures 1, 2) and model membranes (Figure 4).

## 4 Discussion

In this study, we show that the bovine cathelicidin BMAP-28 exerts strongly species-dependent activity against enterococci. *E. faecium* was inhibited at low micromolar concentrations (MIC 5–20 μg/mL) and underwent rapid killing within min, whereas *E. faecalis* remained highly tolerant (MIC ≥80 μg/mL) and displayed little loss of viability under identical conditions. Multiple lines of evidence support membrane disruption as a dominant mechanism in susceptible *E. faecium*, including pronounced surface deformation by SEM and extensive membrane permeabilization by LIVE/DEAD staining. Notably, this species difference could not be overcome by modest analog redesign, suggesting that the primary determinant lies in properties of the bacterial envelope rather than peptide charge or hydrophobicity alone.

Cathelicidins typically act by destabilizing bacterial membranes through cooperative mechanisms such as carpet-like coverage or pore formation (Xhindoli et al., 2016). The rapid multi-log reduction in CFU and high PI uptake observed for *E. faecium* are consistent with fast membrane permeabilization preceding cell death, whereas *E. faecalis* retained viability and morphology under the same conditions. This divergence highlights that membrane-active peptides do not act uniformly across closely related species, and that envelope properties can determine whether peptide binding leads to reversible interaction or irreversible disruption (Nawrocki et al., 2014).

The four BMAP-28 analogs tested here did not substantially improve activity against *E. faecalis*. While AMP optimization often focuses on increasing cationic charge or hydrophobicity to enhance membrane binding (Fjell et al., 2012), our results suggest that such tuning is not the limiting factor in this case. Instead, *E. faecalis* likely presents a membrane environment that restricts productive peptide insertion or dissipates peptide activity. Direct measurements of peptide–membrane binding were not performed here, and future studies using approaches such as circular dichroism or other biophysical assays would help clarify whether binding affinity contributes to the observed species-dependent effects.

Several non-mutually exclusive mechanisms may contribute to *E. faecalis* tolerance, including altered membrane order, differences in phospholipid composition, cell wall architecture, and potential peptide sequestration or degradation (Müller et al., 2016). While our study does not distinguish among these possibilities, our lipid profiling provides a key mechanistic entry point.

A central finding is that *E. faecalis* and *E. faecium* differ reproducibly in membrane fatty acid composition. *E. faecalis* is enriched in saturated fatty acids (C16:0, C18:0), whereas *E. faecium* contains higher levels of unsaturated species (C16:1, C18:1). These differences are expected to alter membrane packing and fluidity, with more saturated membranes forming tighter, less permeable structures and unsaturated membranes being more deformable (Mansilla et al., 2004). This provides a plausible explanation for the observed phenotype, where the more unsaturated *E. faecium* membrane is more susceptible to rapid permeabilization. However, membrane fatty acids alone are unlikely to fully explain the phenotype, as additional factors such as lipid organization and envelope polymers may further modulate peptide activity (Rashid et al., 2017).

Consistent with a membrane-targeting mechanism, BMAP-28 permeabilized PC/PG LUVs, inducing substantial K? leakage at low concentrations. However, vesicles designed to mimic *E. faecalis* and *E. faecium* lipid features exhibited similar leakage profiles, indicating that the simplified differences modeled here are insufficient to reproduce the strong species-dependent effects observed in intact cells (Mishra et al., 2012). Because the PC component was kept constant across vesicle formulations, the present system captures only part of the effect of acyl-chain saturation. This likely reflects limitations of the model system, which lacks key features of Gram-positive envelopes, including peptidoglycan, teichoic acids, membrane proteins, and native lipid complexity (Malanovic and Lohner, 2016; Omardien et al., 2016). Notably, the saturated PG species used in this study (DSPG, C18:0) does not exactly match the dominant saturated fatty acid (C16:0) identified in the bacterial membranes, and future studies using lipids such as DPPG may provide a more physiologically relevant comparison.

Our in-silico analysis suggests that BMAP-28 adopts a predominantly α-helical structure with flexible hinge regions that may facilitate membrane interaction. Such structural adaptability is consistent with membrane-active behavior, enabling dynamic insertion and curvature induction (Wimley, 2010). However, these predictions are based on molecular dynamics simulations and were not experimentally validated under the conditions tested here. Experimental approaches such as circular dichroism spectroscopy using model membranes or lipid extracts from *E. faecalis* and *E. faecium* would provide important validation and further insight into peptide–membrane interactions. Importantly, structural features alone do not explain species specificity. Rather, BMAP-28 appears intrinsically capable of membrane disruption, while the bacterial envelope determines whether this potential is realized—in other words, the peptide is “capable,” but the membrane must be permissive (Epand and Epand, 2009).

These findings have broader implications. First, AMP efficacy can differ substantially even between closely related Gram-positive species, emphasizing the need for species-specific evaluation in antimicrobial development (Khan et al., 2019). Second, membrane fatty acid composition emerges as a meaningful correlation of susceptibility and a useful experimental handle for mechanistic studies.

Several next steps would further clarify the mechanism. Direct measurements of membrane order in *E. faecalis* and *E. faecium* would test whether fatty acid differences translate into distinct physical states. Experimental manipulation of fatty acid composition could help establish causality, while analysis of phospholipid organization and combination treatments with cell wall-active antibiotics may further reveal determinants of tolerance. This study is limited by its reliance on *in vitro* assays and simplified model membranes. While fatty acid composition is associated with susceptibility, causality has not yet been established, and the analog set explored here represents only a limited design space.

In summary, BMAP-28 exhibits pronounced species-dependent activity against enterococci, with rapid membrane permeabilization and killing of *E. faecium* but high tolerance in *E. faecalis*. This difference is associated with distinct membrane fatty acid compositions; however, model membrane experiments indicate that lipid composition alone is insufficient to explain the phenotype. These findings support a membrane-centered framework for cathelicidin activity while highlighting the importance of additional envelope features beyond lipid composition and provide a basis for future peptide optimization strategies against multidrug-resistant Gram-positive pathogens.

## Data Availability

All datasets presented in this study are included in the article. Additional information is available from the corresponding author upon reasonable request.
